# LifeStyle and Exercise Relation to Neck and Back Pain in Saudi Arabia

**DOI:** 10.7759/cureus.32979

**Published:** 2022-12-26

**Authors:** Amal H Almutairi, Amaal M Almalki, Ethar K Alharthi, Zouhor A Alhossaini, Anoud H Alkurayzi, Norah Alharthi, Nuha N Filfilan

**Affiliations:** 1 Medicine, Taif University, Taif, SAU; 2 College of Medicine, Taif University, Taif, SAU; 3 Public Health and Preventive Medicine, Taif University, Taif, SAU

**Keywords:** saudi population, musculoskeletal pain, diet, job, exercise

## Abstract

Background: Personal anguish, incapacity, and a decline in work and life quality are all associated with neck and low back pain, making it a significant socioeconomic burden for individuals and society. It is well known that engaging in regular physical exercise has considerable health benefits.

Objective: The purpose of this research was to investigate the factors contributing to the high rates of musculoskeletal pain experienced by the Saudi Arabian population.

Methods: This population-based, cross-sectional study was done in Saudi Arabia with 2,717 participants aged 18 to 60. A questionnaire was provided online to assess neck, shoulder, and lower back discomfort, time spent in general or aerobic physical activity, time spent sitting, sleep problems, general health, work satisfaction, and nutrition. Using logistic regression, we observed potential risk factors for musculoskeletal pain.

Results: The prevalence of neck pain, shoulder pain, and lower back pain (LBP) were found to be 48.1%, 47.6%, and 63.8%, respectively. It was found that being a female (OR=1.78 [1.41-2.25], p<0.001), married (OR=1.58 [1.34-1.86], p<0.001), and having poor general health status (OR=3.78 [2.2-6.49], p<0.001), sleep disturbances (OR=2.46 [2.04-2.97], p<0.001) and poor job satisfaction (OR=1.29 [1.05-1.60], p=0.016) were independently associated with the prevalence of musculoskeletal pain. The diet of the individuals did not significantly influence the prevalence of MSPs.

Conclusion: Good general health, good sleep, and good job satisfaction were associated with a reduced risk of experiencing neck or shoulder pain, but there was no association between physical activity and MSPs Longitudinal studies are required to acquire a better understanding of the relationship between MSP, aerobic activity, sleep, and diet.

## Introduction

In many countries, neck and upper body musculoskeletal pain (MSP) is a leading source of morbidity and disability. Individuals' physical, social, and psychological well-being can be compromised, resulting in higher healthcare expenditures [1,2]. The prevalence of neck pain in the world's working population is estimated to range between 16.7% to 75.1%, with slightly more prevalence noted among women than men. Several factors contribute to neck and upper body MSP, including ergonomics (physical exhaustion, use of force and vibration, poor posture, repetitive movement), individual characteristics (age, BMI, genetics, history of MSP), lifestyles, and psychosocial characteristics [3,4]. High job strain was associated with upper body pain among males but not among women [5]. Lengthy computer use was also shown to be strongly associated with MSP [6,7]. To prevent neck pain from becoming a long-term condition, it is crucial to identify risk factors and seek treatment early. The prevention, diagnosis, treatment, and management of neck pain can be guided by identifying protective or risk factors, triggers, and outcomes.

Governments face enormous expenditures due to the recurrence and increasing incidence of MSP, which necessitates the implementation of effective new preventive reforms. Numerous studies on MPS have been performed, particularly in developed nations [8-12]. However, few studies have simultaneously investigated neck, shoulder, and low back pain in the Saudi population. Although some studies have explored the pattern and prevalence of MSP in Saudi Arabia, no national-level study has ever been done yet [13,14]. There is no exact data on the prevalence of MSP in Saudi Arabia. From this standpoint, the primary objective of this study is to determine the prevalence of MSP among the Saudi population. Correspondingly, we aimed to analyze the possible risk factors associated with this condition, and we will draw a conclusion by analyzing the impact this condition has on both a person's peoples' physical and social life.

## Materials and methods

A population-based, cross-sectional study was an online survey conducted involving participants from different provinces in Saudi Arabia. The study population included adults aged between 18 years and 60 years (considering the age of retirement in Saudi Arabia). Any individual that is younger than 18 years or older than 60 years of age was excluded. A non-probability sampling technique that used a mixture of convenience and snowball sampling was used to collect responses from the participants based on the eligibility criteria. Twenty-two data collectors, who are medical students at Taif University, helped in distributing the questionnaires. An online pretested self-reported questionnaire was used to collect responses from the participants after obtaining their consent. The data were collected between the period of June to August 2022. On the basis of their relevance and prior identification in the literature as risk factors for neck and shoulder pain, several potentially important factors were assessed and identified.

The questionnaire consisted of three parts (Appendix A). The first part included sociodemographic details (age, gender, province of residence, employment status, occupation type, height, and weight). Part B, the second one, had items that measured the prevalence of neck and shoulder pain, general health status, general state of health, sleep disturbances, aerobic physical activity, time spent sitting, job satisfaction, and time spent working in a week). Responses to the question “Have you suffered any pain in your (body area) within the previous 12 months?” were used to determine the prevalence of lower back pain and neck and shoulder pain. Using a scale from 0 (no pain) to 9 (the greatest pain conceivable) as a reference point. We classified people with back pain into two groups: those with “pain” and “no pain.” In the third part, the dietary intake of each participant was recorded on a dietary chart. This chart had items that recorded the consumption of various food items such as beef, mutton/lamb, chicken, fish, vegetables, fruits, ice cream, chocolates, soft drinks, fruit juice, milk, tea, and coffee. The response options were: (a) Never, (b) 1-2 times/month, (c) once a week, (d) 2-4 times/week, (e) 5-6 times/week, (f) once daily, (g) 2-3 times/day, and (h) ≥ four times/day.

Ethical consideration

Ethical approval for the study was obtained from the research ethics committee of Taif University, Saudi Arabia (IRB No.: 44-069).

A pilot study was done on 20 participants to test the questionnaire for validity and reliability. Expert review and focused group discussion were utilized to evaluate the questionnaire's content and face validity. We did not include the items that had a correlation coefficient of more than 0.70. The test's internal consistency was evaluated (Chronbach's α= 0.714), but the test's test-retest reliability could not be determined due to a lack of available time. Online consent was taken after explaining the purpose and benefits of the study (mentioned as a statement of anonymity and confidentiality at the beginning of the questionnaire).

Statistical analysis and data management

All the collected information was tabulated on a Microsoft Excel sheet and then transferred to IBM Statistical Package for Social Sciences, Version 23 (SPSS Inc., Chicago, IL, USA) for data analysis. Descriptive statistics in the form of frequencies and percentages using suitable tables and figures were used to represent categorical data. Continuous variables were presented using mean and standard deviation. Comparison of continuous variables between categorical variables was evaluated using the Students' “t” test and/or Analysis of variance. Any possible association between categorical variables was measured using Pearson's chi-square test. Multivariate logistic regression was used to analyze key predictive factors for neck and shoulder pain. A p-value <0.05 was considered statistically significant.

## Results

We received around 2,798 responses, of which 2,717 gave consent to participate and completed the total items in the survey. The socio-demographic analysis showed that 2,069 (76.2%) were females, 1,417 (52.2%) belonged to the age group of 18-25 years, 1,621 (59.7%) were single (unmarried), 989 (36.4%) were from the Western Province, 780 (28.7%) were employed, 1,716 (63.2%) had an executive, administrative or clerical duties type of job and 473 (17.4%) were obese (Table 1).

**Table 1:** Sociodemographic details

		N	%
Gender	Female	2069	76.2
	Male	648	23.8
Age (years)	18-25	1417	52.2
	26-35	547	20.1
	36-45	420	15.5
	46-55	239	8.8
	56-60	58	2.1
	>60	36	1.3
Marital status	Single	1621	59.7
	Married	1010	37.2
	Divorced‎/Widow	86	3.2
Province	Central province	652	24.0
	Eastern province	224	8.2
	Northern province	441	16.2
	Southern province	411	15.1
	Western province	989	36.4
Employment status	Employed	780	28.7
	Employed but currently on long leave	24	.9
	Self-employed	115	4.2
	Unemployed	532	19.6
	Retired	95	3.5
	Student	1171	43.1
Job type/nature	Executive, administrative or clerical duties	1716	63.2
	Manual labor or heavy machinery hazardous conditions	163	6.0
	Manual labor or heavy machinery or exposure to certain hazardous conditions	184	6.8
	Skilled or semi-skilled work and not exposed to hazardous conditions	654	24.1
Body Mass Index	Underweight	309	11.4
	Normal	1248	45.9
	Overweight	687	25.3
	Obese	473	17.4

**Figure 1:**
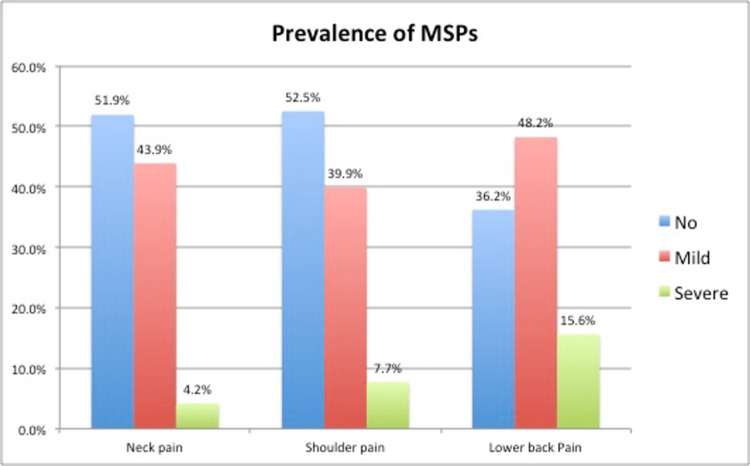
Prevalence of MSPs in study population

The prevalence of neck pain (NP), shoulder pain (SP), and lower back pain (LBP) were found to be 48.1%, 47.6%, and 63.8%, respectively (Figure 1). 

The self-reported physical activities and personal habits are given in Table 2.

**Table 2:** Self-reported physical activity and other personal habits

		N	%
Current general state of health (self reported)	Very poor	9	.3
	Poor	55	2.0
	Good	738	27.2
	Very good	1091	40.2
	Excellent	824	30.3
Sleep disturbances	No	1179	43.4
	Yes, Mild	1286	47.3
	Yes, Severe	252	9.3
Aerobic physical activity per week	Don't do any aerobic physical activity	1015	37.4
	Less than 30 min/week	749	27.6
	30 to 59 min/week	521	19.2
	60 to 89 min/week	179	6.6
	More than 90 min/week	253	9.3
Heavy workouts [Running quickly - cycling quickly - rowing quickly - lifting weights]	Don't do	1758	64.7
	Less than 30 min/week	518	19.1
	30 to 59 min/week	251	9.2
	60 to 89 min/week	79	2.9
	More than 90 min/week	111	4.1
General physical activity per week	Less than 30 min/week	1439	53.0
	30 to 59 min/week	671	24.7
	60 to 89 min/week	257	9.5
	More than 90 min/week	350	12.9
Time spend sitting (per day)	>10 hours	658	24.2
	7-9 hours	806	29.7
	4-6 hours	903	33.2
	< 3 hours	350	12.9
Job satisfaction	Very high	279	10.3
	High	461	17.0
	Moderate	912	33.6
	Low	118	4.3
	Unemployed/none	947	34.9
Time spent working /week	>20 hours/week	1196	44.0
	<20 hours/week	1521	56.0
Smoking status	Current smoker	307	11.3
	Ex-smoker	127	4.7
	Never smoker	2283	84.0

We evaluated the relationship of the prevalence of various MSPs with the participants' sociodemographic, physical activity, and other personal habits (Table 3). It was found that the prevalence of NP, SP, and LBP was significantly higher in females than in males (p<0.001). Three age groups (36-45 years, 46-55 years, and 56-60 years) showed significantly higher prevalence for NP, SP, and LBP (p<0.001). Those who were married reported significantly higher prevalence than others who were single or divorced/widowed (p<0.001). Those who were employed, but on a long-leave, self-employed and retired were found to have a significantly higher prevalence of NP, SP, and LBP (p<0.05). The employment nature didn't show significant differences in the prevalence of NP and LBP, but the prevalence of SP was significantly higher among participants who did manual labor or heavy machinery or exposure to certain hazardous conditions (p=0.035). The prevalence of all three MSPs was significantly higher in participants who reported very poor and poor general health status (p<0.001). It was found that the prevalence of NP, SP, and LBP were significantly higher among participants who reported sleep disturbances either as mild or severe (p<0.001). Participants who had reported doing no aerobic physical activity showed significantly higher prevalence for all three MSPs than others (p<0.05). Similarly, a higher prevalence of MSPs was observed in participants who did not do any heavy workout activity than others (p<0.05). Participants who did general physical activity for <30 minutes showed a significantly higher prevalence of SP and LBP (p<0.05), but NP prevalence didn't show any statistically significant differences (p=0.340). There were no statistically significant differences in the prevalence of all three MSPs observed for time spent sitting/day (p>0.05). The prevalence of all three types of MSPs was significantly higher in participants with lower job satisfaction than others (p<0.001). There was no statistically significant association observed for the prevalence of all three types of MSPs with time spent per week at work (p>0.05). The prevalence of SP was significantly higher in participants who were current smokers (p=0.036). Participants who were overweight and obese (BMI>25) significantly showed a higher prevalence of SP and LBP than others (p<0.05).

**Table 3:** Prevalence of MSPs and their relationship with participants’ sociodemographic characteristics and physical activities

	Neck pain		P value	Shoulder pain			P-value	Lower back pain		P-value
	No	Yes		No	Yes			No	Yes	
Gender										
Female	1008 (48.7%)	1061 (51.3%)	<0.001	997 (48.2%)	1072 (51.8%)		<0.001	694 (33.5%)	1375 (66.5%)	<0.001
Male	402 (62%)	246 (38%)		429 (66.2%)	219 (33.8%)			290 (44.8%)	358 (55.2%)	
Age										
18-25	800 (56.5%)	617 (43.5%)	<0.001	841 (59.4%)	576 (40.6%)		<0.001	613 (43.3%)	804 (56.7%)	<0.001
26-35	289 (52.8%)	258 (47.2%)		270 (49.4%)	277 (50.6%)			179 (32.7%)	368 (67.3%)	
36-45	182 (43.3%)	238 (56.7%)		173 (41.2%)	247 (58.8%)			103 (24.5%)	317 (75.5%)	
46-55	98 (41%)	141 (59%)		97 (40.6%)	142 (59.4%)			67 (28%)	172 (72%)	
56-60	20 (34.5%)	38 (65.5%)		21 (36.2%)	37 (63.8%)			11 (19%)	47 (81%)	
>60	21 (58.3%)	15 (41.7%)		24 (66.7%)	12 (33.3%)			11 (30.6%)	25 (69.4%)	
Marital status										
Single	892 (55%)	729 (45%)	<0.001	933 (57.6%)	688 (42.4%)		<0.001	690 (42.6%)	931 (57.4%)	<0.001
Married	470 (46.5%)	540 (53.5%)		452 (44.8%)	558 (55.2%)			273 (27%)	737 (73%)	
Divorced‎/Widow	48 (55.8%)	38 (44.2%)		41 (47.7%)	45 (52.3%)			21 (24.4%)	65 (75.6%)	
Employment status										
Employed	397 (50.9%)	383 (49.1%)	0.004	370 (47.4%)	410 (52.6%)		<0.001	250 (32.1%)	530 (67.9%)	<0.001
Employed but currently on long leave	9 (37.5%)	15 (62.5%)		11 (45.8%)	13 (54.2%)			8 (33.3%)	16 (66.7%)	
Self-employed	50 (43.5%)	65 (56.5%)		46 (40%)	69 (60%)			37 (32.2%)	78 (67.8%)	
Student	643 54.9%)	528 45.1%)		702 59.9%)	469 40.1%)			502 42.9%)	669 57.1%)	
Retired	36 (37.9%)	59 (62.1%)		43 (45.3%)	52 (54.7%)			27 (28.4%)	68 (71.6%)	
Unemployed	275 (51.7%)	257 (48.3%)		907 (52.9%)	809 (47.1%)			160 (30.1%)	372 (69.9%)	
Employment nature										
Executive, administrative or clerical duties	894 (52.1%)	822 (47.9%)	0.201	907 (52.9%)	809 (47.1%)		0.035	620 (36.1%)	1096 (63.9%)	0.244
Manual labor or heavy machinery hazardous conditions	94 (57.7%)	69 (42.3%)		97 (59.5%)	66 (40.5%)			63 (38.7%)	100 (61.3%)	
Manual labor or heavy machinery or exposure to certain hazardous conditions	85 (46.2%)	99 (53.8%)		81 (44%)	103 (56%)			55 (29.9%)	129 (70.1%)	
Skilled or semi-skilled work and not exposed to hazardous conditions	337 (51.5%)	317 (48.5%)		341 (52.1%)	313 (47.9%)			246 (37.6%)	408 (62.4%)	
General health state										
Excellent	603 (73.2%)	221 (26.8%)	<0.001	590 (71.6%)	234 (28.4%)		<0.001	473 (57.4%)	351 (42.6%)	<0.001
Very good	541 (49.6%)	550 (50.4%)		547 (50.1%)	544 (49.9%)			369 (33.8%)	722 (66.2%)	
Good	251 (34%)	487 (66%)		276 (37.4%)	462 (62.6%)			138 (18.7%)	600 81.3%)	
Poor	15 (27.3%)	40 (72.7%)		12 (21.8%)	43 (78.2%)			4 (7.3%)	51 (92.7%)	
Very poor	0 (0%)	9 (100%)		1 (11.1%)	8 (88.9%)			0 (0%)	9 (100%)	
Sleep disturbances										
No	748 (63.4%)	431 (36.6%)	<0.001	736 (62.4%)	443 (37.6%)		<0.001	542 (46%)	637 (54%)	<0.001
Yes, Mild	590 (45.9%)	696 (54.1%)		613 (47.7%)	673 (52.3%)			392 (30.5%)	894 (69.5%)	
Yes, Severe	72 (28.6%)	180 (71.4%)		77 (30.6%)	175 (69.4%)			50 (19.8%)	202 (80.2%)	
Aerobic physical activity										
Don't do any aerobic physical activity	492 (48.5%)	523 (51.5%)	0.009	505 (49.8%)		510 (50.2%)	0.004	328 (32.3%)	687 (67.7%)	<0.001
Less than 30 min/week	396 (52.9%)	353 (47.1%)		384 (51.3%)		365 (48.7%)		275 (36.7%)	474 (63.3%)	
30 to 59 min/week	276 (53%)	245 (47%)		290 (55.7%)		231 44.3%)		206 (39.5%)	315 (60.5%)	
60 to 89 min/week	92 (51.4%)	87 (48.6%)		90 (50.3%)		89 (49.7%)		61 (34.1%)	118 (65.9%)	
More than 90 min/week	154 (60.9%)	99 (39.1%)		157 (62.1%)		96 (37.9%)		114 (45.1%)	139 (54.9%)	
Heavy workout activity										
Don't do	871 (49.5%)	887 (50.5%)	0.003	880 (50.1%)		878 (49.9%)	<0.001	571 (32.5%)	1187 (67.5%)	<0.001
Less than 30 min/week	277 (53.5%)	241 (46.5%)		277 (53.5%)		241 (46.5%)		219 (42.3%)	299 (57.7%)	
30 to 59 min/week	149 (59.4%)	102 (40.6%)		147 (58.6%)		104 (41.4%)		108 (43%)	143 (57%)	
60 to 89 min/week	43 (54.4%)	36 (45.6%)		44 (55.7%)		35 (44.3%)		30 (38%)	49 (62%)	
More than 90 min/week	70 (63.1%)	41 (36.9%)		78 (70.3%)		33 (29.7%)		56 (50.5%)	55 (49.5%)	
General physical activity										
Less than 30 min/week	739 (51.4%)	700 (48.6%)	0.340	739 (51.4%)		700 (48.6%)	0.049	496 (34.5%)	943 (65.5%)	0.003
30 to 59 min/week	338 (50.4%)	333 (49.6%)		348 (51.9%)		323 (48.1%)		238 (35.5%)	433 (64.5%)	
60 to 89 min/week	137 (53.3%)	120 (46.7%)		131 (51%)		126 (49%)		92 (35.8%)	165 (64.2%)	
More than 90 min/week	196 (56%)	154 (44%)		208 (59.4%)		142 (40.6%)		158 (45.1%)	192 (54.9%)	
Time spend sitting/per day										
>10 hours	341 (51.8%)	317 (48.2%)	0.301	340 (51.7%)		318 (48.3%)	0.863	262 (39.8%)	396 (60.2%)	0.083
7-9 hours	411 (51%)	395 (49%)		426 (52.9%)		380 (47.1%)		270 (33.5%)	536 (66.5%)	
4-6 hours	460 (50.9%)	443 (49.1%)		470 (52%)		433 (48%)		321 (35.5%)	582 (64.5%)	
Less than 3 hours	198 (56.6%)	152 (43.4%)		190 (54.3%)		160 (45.7%)		131 (37.4%)	219 (62.6%)	
Job satisfaction										
Very high	159 (57%)	120 (43%)	<0.001	168 (60.2%)		111 (39.8%)	<0.001	138 (49.5%)	141 (50.5%)	<0.001
High	278 (60.3%)	183 (39.7%)		268 (58.1%)		193 (41.9%)		182 (39.5%)	279 (60.5%)	
Moderate	415 (45.5%)	497 (54.5%)		425 (46.6%)		487 (53.4%)		291 (31.9%)	621 (68.1%)	
Low	52 (44.1%)	66 (55.9%)		59 (50%)		59 (50%)		22 (18.6%)	96 (81.4%)	
None	506 53.4%)	441 46.6%)		506 53.4%)		441 46.6%)		351 37.1%)	596 62.9%)	
Time spend per week at work										
<20 hours/week	800 52.6%)	721 47.4%)	0.907	801 52.7%)		720 47.3%)	0.834	559 36.8%)	962 63.2%)	0.512
>20 hours/week	610 51%)	586 49%)		625 52.3%)		571 47.7%)		425 35.5%)	771 64.5%)	
Smoking status										
Current smoker	143 46.6%)	164 53.4%)	0.097	140 45.6%)		167 54.4%)	0.036	106 34.5%)	201 65.5%)	0.490
Ex-smoker	62 48.8%)	65 51.2%)		69 54.3%)		58 45.7%)		41 32.3%)	86 67.7%)	
Never smoker	1205 (52.8%)	1078 (47.2%)		1217 (53.3%)		1066 (46.7%)		837 (36.7%)	1446 (63.3%)	
Body mass Index										
Underweight	161 (52.1%)	148 (47.9%)	0.054	168 (54.4%)		141 (45.6%)	0.011	136 (44%)	173 (56%)	<0.001
Normal	681 (54.6%)	567 (45.4%)		692 (55.4%)		556 (44.6%)		504 (40.4%)	744 (59.6%)	
Overweight	339 (49.3%)	348 (50.7%)		335 (48.8%)		352 (51.2%)		217 (31.6%)	470 (68.4%)	
Obese	229 (48.4%)	244 (51.6%)		231 (48.8%)		242 (51.2%)		127 (26.8%)	346 (73.2%)	

The Diet Diversity chart of the participants is given in Table 4. The foods were then subgrouped into five categories that are a) Meat diary and products (Beef, Mutton, Chicken, Fish and milk), b) Fruits and Vegetables, c) sweetened and high carb food (Ice cream, chocolates, soft drinks, fruit juice), f) Tea, and e) Coffee. The scores were given based on the frequency of consumption, which ranges from 1 (never) to 8 (>4 times/day). The scores for each food item in each subgroup were added to get a total score for each subgroup, where a higher score indicated increased consumption. We compare the dietary diversity and the prevalence of three types of MSPs. There were no statistically significant differences in dietary scores for Meat, dairy and products, Fruits and Vegetables, and Sweetened and high carb food between participants who had MSPs and those who didn't. At the same time, participants who had increased coffee consumption had a significantly higher prevalence of SP and LBP (P<0.05). Also, LBP prevalence was significantly higher in participants who had consumed more tea.

**Table 4:** Diet diversity chart

	Never	1-2 times/month	Once/week	2-4 times/week	5-6 times/week	Once/day	2-3/day	>4 times/day	Mean (SD)
Beef	1290 (47.5%)	735 (27.1%)	372 (13.7)	245 (9)	34 (1.3)	26 (1)	11 (04)	4 (0.1)	1.95 (1.2)
Mutton	800 (29.4)	866 (31.9)	568 (20.9)	372 (13.7)	53 (2)	38 (1.4)	15 (0.6)	5 (0.2)	2.34 (1.2)
Chicken	127 (4.7)	186 (6.8)	356 (13.1)	1236 (45.5)	375 (13.8)	317 (11.7)	95 (3.5)	25 (0.9)	4.10 (1.4)
Fish	639 (23.5)	1378 (50.7)	436 (16)	179 (6.6)	43 (1.6)	27 (1)	11 (0.4)	4 (0.1)	2.17 (1.0)
Vegetables	146 (5.4)	299 (11)	519 (19.1)	868 (31.9)	351 (12.9)	321 (11.8)	151 (5.6)	62 (2.3)	4.05 (1.6)
Fruit	151 (5.6)	484 (17.8)	649 (23.9)	754 (27.8)	247 (9.1)	262 (9.6)	109 (4)	61 (2.2)	3.73 (1.6)
Ice cream	367 (13.5)	993 (36.5)	671 (24.7)	409 (15.1)	115 (4.2)	102 (3.8)	40 (1.5)	20 (0.7)	2.81 (1.4)
Chocolates	216 (7.9)	502 (18.5)	663 (24.4)	713 (26.2)	239 (8.8)	225 (8.3)	107 (3.9)	52 (1.9)	3.6 (1.6)
Soft Drinks	588 (21.6)	585 (21.5)	545 (20.1)	524 (19.3)	180 (6.6)	154 (5.7)	97 (3.6)	44 (1.6)	3.07 (1.7)
Fruit Juice	401 (14.8)	758 (27.9)	701 (25.8)	501 (18.4)	162 (6)	114 (4.2)	47 (1.7)	33 (1.2)	2.99 (1.5)
Milk	529 (19.5)	567 (20.9)	509 (18.7)	512 (18.8)	190 (7)	271 (10)	94 (3.5)	45 (1.7)	3.25 (1.8)
Tea	357 (13.1)	366 (13.5)	421 (15.5)	600 (22.1)	273 (10)	346 (12.7)	217 (8)	137 (5)	3.98 (2)
Coffee	314 (11.6)	262 (9.6)	337 (12.4)	598 (22)	327 (12)	405 (14.9)	297 (10.9)	177 (6.5)	4.34 (2)

We compare the dietary diversity and the prevalence of three types of MSPs (Table 5). There were no statistically significant differences in dietary scores for Meat, dairy and products, Fruits and Vegetables, and Sweetened and high carb food between participants who had MSPs and those who did not. At the same time, participants who had increased coffee consumption had a significantly higher prevalence of SP and LBP (P<0.05). Also, LBP prevalence was significantly higher in participants who had consumed more tea.

**Table 5:** Prevalence of MSPs based on diet diversity

		Neck pain		P-value	Shoulder pain	P-value	Lower back pain	P-value
Meat diary & products	No		13.9 (3.9)	0.215	13.9 (4.0)	0.297	13.8 (4.0)	0.953
	Yes		13.7 (3.9)		13.7 (3.8)		13.8 (3.9)	
Fruits & Vegetables	No		3.5 (1.3)	0.738	3.5 (1.3)	0.537	3.5 (1.3)	0.629
	Yes		3.5 (1.3)		3.5 (1.3)		3.5 (1.3)	
Sweetened & high carb food	No		5.8 (1.8)	0.120	5.8 (1.8)	0.421	5.7 (1.8)	0.128
	Yes		5.9 (1.8)		5.8 (1.8)		5.8 (1.8)	
Tea	No		4.0 (2.0)	0.972	4.0 (2.0)	0.404	3.9 (2.0)	0.011
	Yes		4.0 (2.0)		4.0 (2.0)		4.1 (2.0)	
Coffee	No		4.3 (2.0)	0.318	4.3 (2.1)	0.021	4.2 (2.1)	0.007
	Yes		4.38 (2.05)		13.9 (4.0)		4.4 (2.0)	

Multivariate logistic regression was done to predict the risk factors and protective factors for MSPs (Table 6). It was found that being a female (OR=1.78 [1.41-2.25], p<0.001), married (OR=1.58 [1.34-1.86], p<0.001), and poor general health status (OR=3.78 [2.2-6.49], p<0.001), sleep disturbances (OR=2.46 (2.04-2.97), p<0.001) and poor job satisfaction (OR=1.29 [1.05-1.60], p=0.016) were independently associated with the prevalence of MSPs.

**Table 6:** Multivariate logistic regression model for MSPs

		B	Std. Error	Wald	P-value	OR	95% Confidence Interval OR	
							Lower Bound	Upper Bound
Gender =Female	-.578	.119	23.534	<0.001	1.78	1.41	2.25
Age >56	.395	.242	2.664	0.103	1.48	0.92	2.38
Marital status= Married	.458	.083	30.495	<0.001	1.58	1.34	1.86
BMI >25	-.028	.120	.055	0.814	0.97	0.77	1.23
General health status = poor/very poor	-1.330	.276	23.240	<0.001	3.78	2.20	6.49
Sleep disturbances = Yes	.899	.096	87.669	<0.001	2.46	2.04	2.97
Aerobic physical exercise= =<30 minutes	-.283	.189	2.233	0.135	0.75	0.52	1.09
Heavy workouts =<30 minutes	-.253	.299	.714	0.398	0.78	0.43	1.40
Sitting hours >6 hours	.119	.091	1.709	0.191	1.13	0.94	1.35
Job satisfaction = poor	-.258	.107	5.794	0.016	1.29	1.05	1.60
	Smoking	-.321	.191	0.635	0.172	1.42	0.87	2.92

## Discussion

The findings of this study showed that female participants, being married, those with poor general health and sleep disturbances, and those with poor job satisfaction were independently associated with developing all three MSPs. Our findings are consistent with previous studies in different parts of the world. Rashid et al. [15] observed that women on medical leave who had both high levels of workplace stress and MSP were significantly less likely to return to work. Chinese researchers Xu et al. observed that those with mental illness, particularly those with depression, were more likely to suffer from chronic MSP than those without mental illness [16]. Hallman et al. found that women who regularly engaged in intense physical activity were more likely to have a worsening of their preexisting NP over time. Also, walking, running, or cycling, were associated with lowering NP severity with no major gender differences [17]. Other factors like age, obesity, limited musculoskeletal function, higher cognitive work demands, poor working atmosphere, low social support, heavy physical workload, lack of autonomy, and insufficient physical activity all have a negative impact on the work abilities of long-term MSP patients [18,19]. Keeping active and exercising for at least 150 minutes per week at a moderate intensity or 75 minutes per week at a strong intensity is one of the important things individuals can do for their health. Additional health benefits can be obtained from engaging in moderate physical activity for up to five hours per week or intense exercises for up to 2.5 hours per week [20].

It is widely acknowledged that a lack of exercise contributes to NP and poor health [20-22]. Still, it appears that there are substantial differences in the way these factors interact among different people [22]. Cardiovascular diseases, cancer, diabetes, and poor sleep are just some of the medical problems that have been found to improve with physical activity [20]. The majority of our participants reported having relatively modest levels of overall physical activity. However, it was not possible to draw any definite conclusions about cause and effect in the present investigation. For instance, those who engaged in more vigorous exercise may have experienced less NP, and those who did not may have been more willing to perform moderate to vigorous exercises. A lower incidence of NP was found in a previous study [21] when participants engaged in physical exercise for at least five hours per week; however, the intensity level of the activity was not specifically described in that study.

The present research found that all three types of MSPs were associated with disrupted sleep. Our findings support the previous pieces of evidence that reported an association between sleep disturbances and MSPs. It has been previously observed by Holm et al. [23] and Finan et al. [24] that those who report poor job performance and sleep disturbances have a greater chance of acquiring neck and lower back pain. However, it is not clear whether sleep disturbances lead to MSPs or MSPs related to sleep disturbances. Evidence shows that Insomnia is a common complaint among those who live with chronic pain [25]. In light of this growing body of research, it seems reasonable to conclude that sleep deprivation may contribute to the onset of musculoskeletal discomfort. According to past research [23], shorter sitting hours are associated with less likelihood of developing neck and back discomfort. In contrast, we detected no statistically significant association between sitting time spent and the prevalence of MSPs. Pain might be the result of doing the same thing over and over again without taking sufficient sitting breaks [26] or from stress [27]. One further study found that blue-collar employees who sat more during the workday had a lower likelihood of experiencing NP [28]. Therefore, it is likely that there is a great deal of individual variation in the amount of time spent sitting and relaxing in relation to the physical demands placed on muscles.

Numerous clinical and experimental studies have demonstrated that cigarette smoking negatively impacts the musculoskeletal system and negatively impacts the outcome of a variety of orthopedic illnesses and surgical interventions [29,30]. Our findings showed that smokers had a significantly higher prevalence of SP, but there was no association observed between NP and LBP. The therapeutic importance of this secondary but crucial problem is often neglected, despite the fact that the number of smokers is still high across the world. Since smoking has been linked to an increase in negative psychological effects, it was hypothesized that people living with chronic MSP would be driven to smoke because they believe it will help them deal with the pain they are experiencing. However, some studies [31,32] have disproved this theory by showing that the relationship persists even after controlling anxiety and mood disorders. In the current research, BMI > 25 was found to have experienced more LBP and NP than others. There is some evidence to suggest that obesity has both biomechanical and meta-inflammatory impacts on the spine [33]. On the other hand, body mass index (BMI) measures of obesity are not a precise indicator of adiposity since they do not distinguish between fatty and lean mass. Furthermore, males and females have distinct body compositions [34,35], with women having a larger fat content. A meta-analysis found that being overweight or obese is associated with LBP more strongly in females than in males [36]. Although biomechanical parameters linked to spinal loading are associated with MSPs, particularly LBP, our findings suggest that a systemic metabolic mechanism associated with excess adipose tissue may potentially play a role in back pain and impairment.

Our study findings did not show a significant impact of diet on MSPs. Fruits, vegetables, and whole grains are among the foods suggested to be effective for easing MSPs [37]. Omega-3 fatty acids are found in fish oil [38], olive oil [39], turmeric [40], and green tea [41]; resveratrol is found in grapes and wine [42], capsaicin is found in pepper, and various flavonoids found in cabbage [43], cocoa [44], apple [45], and citrus fruits [46] have all been evaluated for their anti-inflammatory effects and/or their therapeutic effect on MSPs pain. Chronic inflammation and oxidative stress [47] are the primary determinants of chronic pain [37], and studies evaluating the benefits of nutritional interventions suggest that some foods may have anti-inflammatory activities, neutralizing these processes. There is evidence that some diets can modulate the immune system and pain perception, hence reducing functional loss due to musculoskeletal disorders and enhancing the quality of life [37,48]. However, the mechanisms behind these interactions are still unknown, and more research is needed [49] since various foods have distinct qualities and methods that operate to alleviate pain and other musculoskeletal problems.

Due to the cross-sectional design of our research, we were unable to establish any causative relationships between the various factors and MSPs. This is the primary limitation of our study. Self-reported estimates of exercise activity levels, general health condition, and diet have also been questioned, which is another limitation. To test these characteristics objectively in every single individual who is a participant in a large population-based cohort with many participants would be incredibly expensive and possibly not even practicable.

## Conclusions

The current study found that female gender, married, poor general health status, poor sleep quality, and poor job satisfaction were associated with MSPs. However, no significant association was observed between different foods consumed and MSPs. More high-quality studies are needed to evaluate the relationship between MSP and diet. Insights into the relationship between upper body pain, physical activity, sleep, and health may be useful in developing effective ways to prevent and manage neck, shoulder, and lower back pain. Despite the negative effects of smoking on osteoarthritis, rheumatoid arthritis, intervertebral disc degeneration, reduced muscle mass and strength, muscular discomfort, tendons degeneration, and ruptures, smoking was not independently associated with MSP in our study. Some individuals may have quit smoking due to health concerns, but tobacco smoke may also have altered their pain perception threshold. Initiatives to encourage healthy habits should be an organizational policy priority to assist employees in staying healthy and coping with work, especially given the relatively high incidence of unhealthy lifestyles in the general working population. In order to fully comprehend the relationships between MSP and health, sleep difficulties, and aerobic activity, long-term longitudinal studies are warranted.
